# The Prognostic Impact of the Cachexia Index in Patients Hospitalized with Heart Failure

**DOI:** 10.3390/medicina62071246

**Published:** 2026-06-27

**Authors:** Vahit Can Cavdar, Hidayet Ozan Arabaci, Zafer Guven, Emine Meltem, Hatice Ozkul, Ayse Satilmisoglu, Kader Onay, Elif Kilic Dinler, Ismail Can Ciftci, Yalcin Gokmen, Mert Aric, Ayli Heydari, Cagdas Kaya, Veysi Kapagan, Eser Onur Cakir, Ahmet Oz

**Affiliations:** 1 Department of Internal Medicine, Istanbul Training and Research Hospital, University of Health Sciences, 34096 Istanbul, Turkey; dr.haticegl@hotmail.com (H.O.); aysesatilmisoglu@gmail.com (A.S.); kaderonayy@hotmail.com (K.O.); elifcankilic@gmail.com (E.K.D.); yalcin_94@hotmail.com (Y.G.); ayliheydari@gmail.com (A.H.); cagdaskaya54@gmail.com (C.K.); veysikapagan@gmail.com (V.K.); eseronurcakir@gmail.com (E.O.C.); 2 Department of Cardiology, Yuksekova State Hospital, 30300 Hakkari, Turkey; drhidayetozanarabaci@gmail.com; 3 Deparment of Cardiology, Gaziosmanpasa Training and Research Hospital, 34255 Istanbul, Turkey; drzaferguven80@gmail.com; 4 Department of Radiology, Istanbul Training and Research Hospital, University of Health Sciences, 34668 Istanbul, Turkey; meltem_emine@hotmail.com; 5 Department of Internal Medicine, Beylikduzu State Hospital, 34500 Istanbul, Turkey; mert_aric@hotmail.com; 6 Department of Cardiology, Istanbul Training and Research Hospital, University of Health Sciences, 34098 Istanbul, Turkey; drozahmet@gmail.com

**Keywords:** heart failure, skeletal muscle index, cachexia index, prognosis, mortality

## Abstract

*Background and Objectives*: Cachexia is a systemic wasting syndrome associated with poor outcomes in chronic diseases, including heart failure (HF). Although the cachexia index (CXI), which integrates skeletal muscle mass, serum albumin, and the neutrophil-to-lymphocyte ratio, has shown prognostic value in oncology, its clinical significance in HF remains poorly defined. *Materials and Methods*: This retrospective single-center cohort study evaluated a selected subgroup of adults hospitalized with decompensated heart failure between January 2020 and January 2025 who had undergone abdominal computed tomography within the preceding 6 months, enabling CT-based body composition assessment. Skeletal muscle index was measured at the L3 vertebral level, and CXI was calculated as (SMI × serum albumin)/neutrophil-to-lymphocyte ratio. Patients were followed for all-cause mortality and HF-related rehospitalizations. *Results*: A total of 127 patients were included (mean age 70.45 ± 12.73 years; 51.2% male). CXI showed excellent discrimination for mortality (AUC 0.951; 95% CI 0.905–0.996), with an optimal cut-off value of <20.87. Patients with low CXI had significantly higher all-cause mortality (80.5% vs. 4.7%, *p* < 0.001) and more HF-related hospitalizations [4 (3–5) vs. 0.5 (0–1), *p* < 0.001] than those with high CXI. *Conclusions*: In patients hospitalized with decompensated HF, low CXI was strongly associated with all-cause mortality and recurrent hospitalization, suggesting that CXI may serve as an integrative prognostic marker in this population.

## 1. Introduction

Loss of lean body mass, weakness, and weight loss are hallmark features of cachexia, a progressive and systemic wasting syndrome that frequently develops during the course of illnesses such as cancer, organ failure, or infections. It is one of the most challenging systemic response disorders and a major contributor to morbidity and mortality [1].

Although cachexia and its clinical effects are most prominently observed in solid organ cancers, end-stage renal failure, rheumatologic diseases, chronic obstructive pulmonary disease, acquired immune deficiency syndrome, and sepsis, it is also recognized as a clinical feature of chronic heart failure, where it significantly contributes to morbidity and mortality [2,3,4].

Heart failure (HF) is a major public health concern affecting over 6 million individuals in the United States. When decompensation occurs, hospital admissions often become inevitable, posing serious health risks to patients and placing a substantial financial burden on the healthcare system. Although clinical studies have identified effective treatment strategies, hospitalization and mortality rates associated with HF remain high [5].

Various biomarkers and predictive indices have been developed to estimate prognosis in HF. In this context, nutritional assessment tools have emerged as valuable instruments for evaluating disease trajectory and prognostic outcomes in patients with HF [6]. Various risk scoring systems have also been developed to assess the short-term risk of adverse events following acute HF. When interpreted together with the patient’s clinical condition, these scores provide valuable guidance in predicting the clinical course [7].

The cachexia index, an emerging biomarker that combines fat-free mass measurements with inflammatory and metabolic markers, has gained traction across oncology settings due to its robust prognostic utility [8]. Lower index values have consistently been linked to reduced survival in several malignancies, particularly gastrointestinal cancers, and have demonstrated predictive value for the future development of cancer-associated cachexia [8,9,10,11]. Yet, despite its growing relevance, the prognostic implications of the cachexia index in HF remain poorly defined, and evidence evaluating its association with rehospitalization and mortality in this population is exceedingly limited.

Accordingly, the present study aimed to investigate the impact of the cachexia index on recurrent hospitalizations and all-cause mortality in a selected cohort of patients hospitalized with heart failure who had available CT-based skeletal muscle assessment. By doing so, we sought to contribute to the evolving understanding of body composition–based risk stratification in HF and to explore the potential role of the cachexia index as a novel, clinically informative prognostic tool within this imaging-defined population.

## 2. Materials and Methods

### 2.1. Study Design and Population

This retrospective, single-center cohort study included adults admitted to the Coronary Care Unit (CCU) of Istanbul Training and Research Hospital with a diagnosis of decompensated HF between January 2020 and January 2025. All clinical, laboratory, and imaging data were extracted from institutional electronic medical records and national health databases. Detailed patient information, including demographic variables, HF subtype, comorbid conditions, prior coronary interventions, medication history, biochemical parameters, echocardiographic findings at presentation, number of prior HF-related CCU admissions, and mortality status, was systematically collected.

Because inclusion required the availability of abdominal CT imaging suitable for skeletal muscle quantification, the study population represents a selected subgroup of hospitalized HF patients rather than an unselected real-world HF cohort.

A total of 3434 patients were screened during the study period, and 127 were ultimately included after the application of strict eligibility criteria. Exclusion criteria were as follows: age < 18 years; HF attributable to pulmonary embolism; confirmed severe pulmonary arterial hypertension (PAH); acute decompensation triggered by PAH crisis; cirrhosis; end-stage renal disease; advanced HF with left ventricular ejection fraction (LVEF) < 20%; pulmonary hypertension treated with ≥3 PAH-specific agents or any parenteral PAH therapy; active malignancy or malignancy diagnosed during follow-up; and absence of an abdominal computed tomography scan within the preceding 6 months.

Severe PAH was defined as pulmonary vascular resistance > 5 Wood units or mean pulmonary arterial pressure > 30 mmHg confirmed by right heart catheterization [12]. End-stage renal disease was defined as a glomerular filtration rate < 15 mL/min/1.73 m^2^ or chronic dialysis dependence. Advanced HF was defined according to European Society of Cardiology criteria as persistent functional impairment with LVEF ≤ 20% despite optimal therapy [13]. Coronary artery disease was defined as ≥50% stenosis on angiography or previous percutaneous coronary intervention or coronary artery bypass grafting surgery.

**Figure 1:**
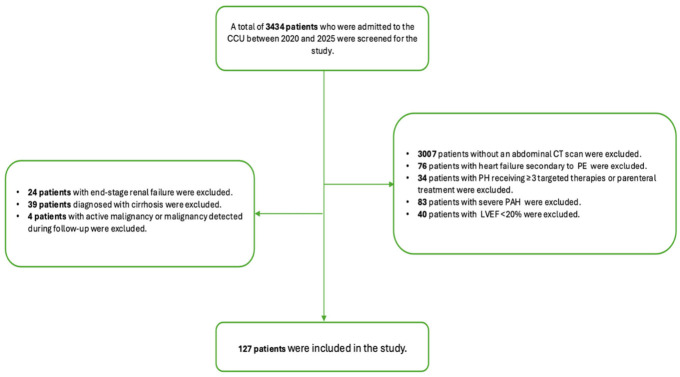
Flowchart of the study. CT: computed tomography, CCU: coronary care unit, LVEF: left ventricular ejection fraction, PAH: pulmonary arterial hypertension, PE: pulmonary embolism, PH: pulmonary hypertension.

The inclusion criteria consisted of adults aged ≥18 years who had undergone an abdominal CT scan for any indication within the preceding 6 months. The patient selection process is summarized in Figure 1.

Patients who met the inclusion criteria were followed from January 2020 to January 2025, and the study endpoints were defined as all-cause mortality and heart failure-related rehospitalizations occurring during the follow-up period.

### 2.2. Data Collection and Baseline Characteristics

Demographic and clinical characteristics, including hypertension, diabetes mellitus, atrial fibrillation, peripheral arterial disease, chronic kidney disease, previous myocardial infarction, and history of revascularization, were retrieved from electronic hospital records and the national health system database. Medication profiles were verified through pharmacy records and outpatient charts. Laboratory findings and echocardiographic parameters were recorded from the initial CCU admission during the study period. All data were reviewed independently to ensure consistency and accuracy. A total of 3434 patients were screened during the study period. After applying the predefined inclusion and exclusion criteria, 127 patients (3.7%) were eligible and included in the final analysis.

### 2.3. Assessment of Skeletal Muscle Mass

Skeletal muscle mass was evaluated using abdominal CT scans previously obtained for clinical purposes. No additional imaging was performed. Skeletal muscle index (SMI) was calculated as follows:

*SMI* (cm^2^/m^2^) = *total cross-sectional skeletal muscle area* (cm^2^)/*height*^2^ (m^2^).

CT imaging was performed using either a 64-slice Toshiba Aquilion (Toshiba Medical Systems, Tochigi, Japan) or a 128-slice Philips Brilliance (Philips Healthcare, Amsterdam, The Netherlands) scanner (slice thickness 0.4 mm, 120 kVp, 80 mAs). Images were reconstructed in axial, sagittal, and coronal planes and transferred in DICOM format to a workstation (SimplePACS v2999-20210401, Izmir, Turkey). The cross-sectional muscle area was automatically segmented at the level of the L3 vertebra, followed by manual correction when segmentation extended beyond muscle boundaries. All CT assessments were performed by a radiologist blinded to clinical variables and outcomes.

### 2.4. Cachexia Index Determination

The cachexia index (CXI)—a composite marker integrating muscle mass, nutritional status, and systemic inflammation—was calculated using the following equation:

CXI = (SMI × serum albumin)/neutrophil-to-lymphocyte ratio (NLR).

SMI was expressed in cm^2^/m^2^, serum albumin in g/dL, and NLR was derived from complete blood count parameters [14]. Patients were categorized into low- and high-CXI groups. Mortality rates and HF-related CCU admissions were compared between these categories.

Ethics approval was obtained from the Ethics Committee of the University of Health Sciences, Turkey, İstanbul Training and Research Hospital (Date: 2 May 2025, No: 101). All procedures performed in this study were in accordance with the ethical standards of the institutional research committee and with the 1964 Declaration of Helsinki and its later amendments.

Written informed consent was waived by the Institutional Ethics Committee because of the retrospective design of the study and the use of de-identified clinical data.

### 2.5. Statistical Analysis

Normality of distribution for continuous variables was assessed using the Shapiro–Wilk test. Normally distributed data were expressed as mean ± standard deviation (SD) and compared using the independent-samples *t*-test. Non-normally distributed variables were presented as median and interquartile range (Q1–Q3) and analyzed using the Mann–Whitney U test. Categorical variables were summarized as counts and percentages and compared using Pearson’s chi-square test or Fisher’s exact test, as appropriate. The prognostic performance of the cachexia index for predicting mortality was assessed using receiver operating characteristic (ROC) curve analysis, with the optimal cut-off derived from the Youden index. Associations between mortality and demographic, clinical, laboratory, and therapeutic variables were first evaluated by univariate logistic regression. Variables demonstrating significance were entered into a multivariate logistic regression model using a stepwise (enter) method. Components of the cachexia index—neutrophil count, lymphocyte count, NLR, and SMI—were excluded from multivariate modeling to avoid collinearity. All statistical analyses were performed using SPSS version 25.0. A two-sided *p*-value < 0.05 was considered statistically significant.

## 3. Results

A total of 127 patients were included in the study, with a mean age of 70.45 ± 12.73 years. Of these, 65 (51.2%) were male and 62 (48.8%) were female. The comorbidities of the study population by gender are summarized in Table 1. When patients were stratified by gender and cardiac function was compared between the groups, ejection fraction was significantly higher in females (*p* < 0.001). In contrast, males had significantly larger LVEDd and LA diameters (*p* = 0.001 and *p* = 0.042, respectively), as shown in Table 2. On evaluation of the laboratory findings, females were found to have significantly lower hemoglobin, hematocrit, and eGFR values (*p* < 0.001 for all). No significant sex-related differences were observed in other hematological and biochemical parameters, as summarized in Table 2.

SMI was significantly higher in male patients (*p* < 0.001). Regarding treatment strategies, the use of diuretics and ivabradine was significantly more frequent in males (*p* = 0.029 and *p* = 0.045, respectively). No significant difference in mortality was observed between males and females, as presented in Table 1.

**Figure 2:**
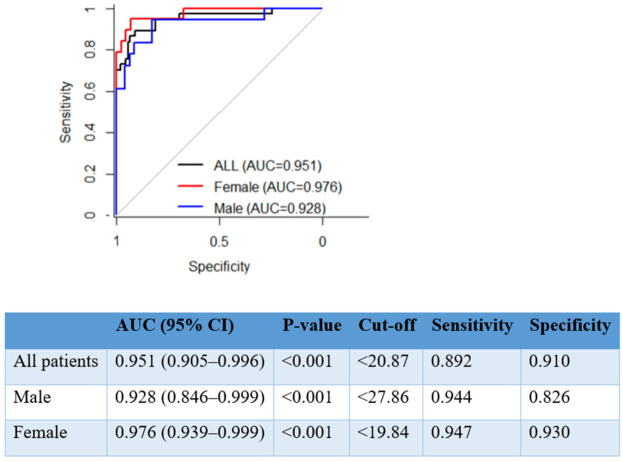
ROC curve analysis of the cachexia index in males and females. AUC: area under the receiver operating characteristic curve; CI: confidence interval. The gray diagonal line represents the reference line (no-discrimination line, AUC = 0.5).

The predictive power of the CXI for mortality was high in the overall study population, with the AUC reaching statistical significance (AUC = 0.951; 95% CI 0.905–0.996; *p* < 0.001). The identified cut-off value was <20.87, yielding a sensitivity of 89.2% and a specificity of 91.0%. In gender-specific subgroup analyses, the AUC for CXI in males was 0.928. The cut-off value in males was <27.86, with a sensitivity of 94.4% and a specificity of 82.6%. In females, the predictive power of CXI was higher, with an AUC of 0.976. The cut-off value for females was <19.84, corresponding to a sensitivity of 94.7% and a specificity of 93.0%, as shown in Figure 2.

When patients were stratified into low- and high-CXI groups, the prevalence of diabetes was significantly lower in the low-CXI group (*p* = 0.032). Analysis of medication use revealed that SGLT-2 inhibitor therapy was significantly less frequent in patients with a low CXI, as presented in Table 3 (*p* = 0.044). Patients with a low CXI exhibited significantly higher WBC and neutrophil counts, NLR, CRP, and LDH levels. In contrast, lymphocyte counts and SMI were significantly lower in the low-CXI group, as presented in Table 3. Patients with a low CXI had a significantly higher number of hospitalizations due to heart failure (*p* < 0.001). In addition, all-cause mortality was significantly higher in the low-CXI group, as shown in Table 3 (*p* < 0.001).

When patients were stratified into survivors and non-survivors, those in the non-survivor group exhibited significantly higher WBC and neutrophil counts, NLR, and CRP levels. Conversely, lymphocyte counts were significantly lower in the non-survivor group, as summarized in Table 4.

Low hematocrit (HTC) levels (<33%; 62.2% vs. 41.1%; *p* = 0.031), low SMI (<40; 89.2% vs. 32.2%; *p* < 0.001), and multiple hospitalizations due to heart failure (99.7% vs. 52.2%; *p* < 0.001) were found to be significantly associated with mortality, as shown in Table 4.

In univariate logistic regression analysis, a low CXI was identified as a significant and strong risk factor for mortality (*p* < 0.001). Low hematocrit levels (<33%) were also significantly associated with mortality (*p* = 0.048). Additionally, WBC count (*p* = 0.015), neutrophil count (*p* < 0.001), lymphocyte count (*p* < 0.001), NLR (*p* < 0.001), and CRP levels (*p* = 0.005) were all identified as statistically significant risk factors for mortality, as presented in Table 5.

In stepwise multivariate logistic regression analysis, a low CXI was found to be independently and significantly associated with mortality (OR = 98.97; 95% CI 23.23–421.69; *p* < 0.001). Additionally, low hematocrit levels (<33%) were independently associated with mortality (OR = 3.98; 95% CI 1.00–16.34; *p* = 0.048). These findings indicate that both CXI and hematocrit were independent risk factors for predicting mortality, as presented in Table 5.

## 4. Discussion

In this retrospective cohort of a selected subgroup of patients hospitalized with decompensated heart failure who had available CT-based body composition assessment, we found that a low cachexia index was strongly associated with adverse clinical outcomes, particularly all-cause mortality and recurrent heart failure hospitalizations.

The study included 127 patients with a mean age of 70.45 ± 12.73 years, and CXI showed excellent discrimination for all-cause mortality in the overall cohort, with an AUC of 0.951 (95% CI 0.905–0.996), an optimal cut-off of <20.87, 89.2% sensitivity, and 91.0% specificity. In sex-specific analyses, the AUC remained high in both men and women and was numerically higher in women (0.928 in men and 0.976 in women). Low CXI also remained independently associated with mortality in multivariable analysis (OR 98.97, 95% CI 23.23–421.69, *p* < 0.001), while low hematocrit (<33%) emerged as another independent predictor (OR 3.98, 95% CI 1.00–16.34, *p* = 0.048). These findings support the concept that CXI may function as an integrated marker of systemic vulnerability in hospitalized HF, rather than merely reflecting conventional measures of cardiac dysfunction.

Several limitations should be considered when interpreting our findings. First, only 127 of the 3434 screened patients were included in the final analysis. This high exclusion rate may have introduced selection bias and spectrum bias, resulting in a study population that may not fully represent the broader patient population encountered in routine clinical practice. Consequently, the generalizability of our findings may be limited.

An important methodological feature of the present study is the selected nature of the study population. We deliberately excluded conditions in which cachexia, sarcopenia, and skeletal muscle wasting are highly prevalent and may independently dominate prognosis, including advanced pulmonary arterial hypertension, end-stage renal disease, cirrhosis, active malignancy, and advanced HF [15,16]. This strategy likely reduced contamination by competing wasting syndromes and allowed a cleaner evaluation of the prognostic signal of CXI in HF itself. This decision is biologically and clinically justifiable, as cachexia and sarcopenia are well recognized not only in HF but also in cirrhosis, chronic kidney disease, pulmonary arterial hypertension, and malignant diseases, where they substantially influence functional status and survival [17,18,19]. Accordingly, although these exclusions limit generalizability, they likely improved the internal validity of the present analysis.

Another important observation in our study is that the prognostic signal captured by CXI appeared to extend beyond several conventional cardiac parameters. Although patients with low CXI tended to have lower LVEF and higher NT-proBNP levels, these differences were not statistically significant. Similarly, LVEF category, atrial fibrillation, coronary artery disease, and natriuretic peptide thresholds were not significantly associated with mortality in our cohort. This should be interpreted cautiously, as a large body of evidence has consistently demonstrated the prognostic relevance of traditional cardiovascular markers, including LVEF, natriuretic peptides, and major cardiac comorbidities, in patients with heart failure [20,21,22,23]. Therefore, the absence of statistical significance in our study likely reflects the relatively small sample size, single-center design, and selected study population rather than a lack of clinical importance of these established parameters. In this context, our findings more appropriately suggest that CXI may capture an additional dimension of risk related to frailty, inflammatory-catabolic stress, and reduced physiological reserve, which may not be fully reflected by conventional cardiac markers alone.

The prognostic behavior of CXI in our cohort is also consistent with prior oncology literature, where this index was originally developed and validated. Previous studies in advanced non-small cell lung cancer, small cell lung cancer, diffuse large B-cell lymphoma, and other malignancies have shown that low CXI is associated with poorer survival, reduced treatment tolerance, and more pronounced inflammatory and nutritional impairment [9,14,24]. More recent systematic reviews have confirmed the adverse prognostic implications of low CXI across malignant diseases [25]. Our findings suggest that this prognostic concept is not confined to cancer, but may be generalizable to other chronic catabolic-inflammatory states such as HF. This cross-disease consistency further strengthens the biological plausibility of CXI as a marker of systemic wasting and reduced physiologic reserve.

The biological relevance of CXI in HF appears highly plausible. By incorporating skeletal muscle index, serum albumin, and neutrophil-to-lymphocyte ratio (NLR), CXI captures three complementary yet interrelated domains: muscle mass, nutritional reserve, and systemic inflammation. This integrated structure is biologically attractive in HF, where muscle wasting, malnutrition, inflammatory activation, and catabolic stress frequently coexist and interact [26]. Albumin is a multifactorial biomarker influenced not only by nutritional status but also by inflammation, hepatic function, and fluid status. Therefore, low albumin levels may reflect systemic disease severity in addition to malnutrition in HF patients.

In our cohort, patients with low CXI had lower SMI and a distinctly more inflammatory profile, characterized by higher WBC count, neutrophil count, NLR, CRP, and LDH. This pattern supports the concept that low CXI identifies a catabolic-inflammatory phenotype in HF. Previous HF studies have usually focused on one component at a time, such as sarcopenia, hypoalbuminemia, or inflammatory markers like NLR.

Lopez et al. showed that low skeletal muscle mass measured on CT during an acute HF hospitalization was independently associated with increased late mortality in a cohort of 160 HF patients [27]. Matsumura et al. subsequently demonstrated in 210 older patients with acute decompensated HF that reduced psoas muscle mass predicted cardiac death and that this relationship appeared particularly relevant in HFpEF [28]. Konishi et al. further reported that, in older HF patients, sarcopenia contributed to mortality similarly in HFpEF and HFrEF [29]. Our study is concordant with these observations but adds an important layer: rather than relying on muscle mass alone, we show that a composite metric incorporating SMI, albumin, and NLR has very high discriminatory performance for mortality. This may explain why, in our cohort, low CXI separated risk even though EF distributions were not significantly different between low- and high-CXI groups and NT-proBNP values were only numerically higher in the low-CXI group.

The nutritional component of CXI is also clinically meaningful in HF. Malnutrition has increasingly been recognized as a major determinant of outcomes in hospitalized decompensated HF, and a recent systematic review and meta-analysis found that higher nutritional risk was associated with poorer prognosis and higher mortality in this setting [6]. Albumin, in turn, has repeatedly been linked to adverse outcomes in HF. A meta-analysis of 16,763 HF patients demonstrated that hypoalbuminemia was associated with increased in-hospital mortality and long-term mortality in acute HF, as well as substantially higher long-term mortality in chronic HF [30]. Another meta-analysis also supports an association between hypoalbuminemia and poor prognosis, especially in acute HF [31]. At the same time, more recent reviews emphasize that albumin should be interpreted not simply as a nutritional marker, but also as a surrogate for chronic congestion, inflammation, renal dysfunction, and multisystem disease severity [32]. Our findings are consistent with this framework, suggesting that the strong prognostic performance of CXI, including its excellent discrimination for mortality in our cohort (AUC 0.951), is likely related to the combined assessment of albumin, SMI, and NLR rather than albumin alone.

The inflammatory profile of the low-CXI group provides additional biological support for the prognostic signal observed in our study. Patients with low CXI had significantly higher WBC and neutrophil counts, higher NLR, higher CRP, and higher LDH, whereas lymphocyte counts and SMI were lower, indicating that low CXI tracked a state of heightened inflammation and depleted physiologic reserve. This is highly consistent with the HF literature, showing that inflammatory activation is not merely epiphenomenal but also prognostically meaningful. Meta-analyses and cohort studies have shown that elevated NLR is associated with worse outcomes in both acute and chronic HF [33,34]. In a 2023 systematic review and meta-analysis including 36 studies and 18,231 HF patients, elevated NLR was associated with higher mortality risk, and NLR was significantly higher in deceased than in surviving patients [35]. Therefore, the strong performance of CXI in our cohort likely reflects its ability to embed inflammatory burden within a broader metabolic and body-composition framework, rather than functioning as a muscle or nutrition marker alone.

A particularly noteworthy aspect of this study is that low CXI was associated not only with mortality but also with a heavier burden of HF rehospitalization. This is clinically relevant because recurrent HF hospitalization reflects disease instability, persistent congestion, frailty, and high healthcare utilization. In our results, low-CXI patients had significantly more HF admissions, reinforcing the concept that CXI is not simply a terminal prognostic marker but may also capture a trajectory of repeated decompensation. This expands the potential clinical utility of CXI beyond survival estimation and suggests that it may be useful for identifying patients who require closer post-discharge monitoring, more intensive nutritional assessment, and earlier multidisciplinary intervention.

Sex-related findings in our study should be interpreted cautiously. Although women were older and had higher EF, whereas men had larger cardiac dimensions and greater ischemic burden, CXI values were similar across sexes, and its predictive performance for mortality was numerically higher in women (AUC 0.976 vs. 0.928). This observation is intriguing because women with HF more commonly exhibit HFpEF-dominant phenotypes, and prior work has shown that sarcopenia contributes to mortality in HFpEF as well as HFrEF [28,29]. This difference may reflect sex-related variation in body composition, inflammatory burden, or HF phenotype, including the possibility that CXI captures systemic vulnerability particularly well in women with relatively preserved EF. However, given the modest sample size and subgroup nature of the analysis, this finding should be considered hypothesis-generating rather than definitive.

Another clinically meaningful result of our study is the independent association between low hematocrit and mortality. In HF, anemia and low hematocrit may result from hemodilution, renal dysfunction, chronic inflammation, iron deficiency, impaired erythropoiesis, or malnutrition. A recent systematic review and meta-analysis of patients with acute heart failure showed that anemia was associated with higher risks of both all-cause mortality and all-cause HF events, including short-term and 1-year mortality [36]. Earlier work in hospitalized HF patients with cardiac cachexia also suggested that hemoglobin is among the pivotal prognostic markers in this phenotype [37]. In our analysis, low hematocrit remained independently associated with mortality after adjustment, suggesting that impaired hematologic reserve contributes additional risk beyond CXI itself. In this context, low hematocrit likely reflects more than simple anemia; rather, it may represent the combined effects of systemic disease severity, inflammatory activation, reduced nutritional reserve, and chronic congestion. Taken together, low CXI and low hematocrit may therefore identify a subgroup with especially advanced systemic compromise.

This study has several limitations. First, it was retrospective and conducted at a single center, which inherently limits external validity. The sample size was relatively small, and only 127 of 3434 screened patients were ultimately included after strict eligibility criteria were applied. İnclusion required an abdominal CT within the preceding 6 months, which may have introduced selection bias and may also limit applicability to broader HF populations that do not routinely undergo CT-based body composition assessment. This requirement resulted in a highly selected imaging-based cohort and likely introduced substantial selection bias, as patients undergoing abdominal CT may systematically differ from the broader real-world heart failure population. The exclusions that strengthened internal validity by reducing confounding from severe non-cardiac wasting syndromes also limit generalizability to highly multimorbid real-world HF cohorts. The cut-offs used for ROC discrimination were derived and tested in the same cohort, which may have inflated the apparent diagnostic performance of CXI. Conventional cachexia-related clinical variables such as unintentional weight loss, appetite status, frailty, and functional capacity were not systematically available because of the retrospective design. Given the relatively small number of mortality events, the multivariable model may have been susceptible to overfitting and unstable effect estimates, particularly regarding the very high odds ratio observed for low CXI. The primary analyses were performed using a pooled cohort threshold because of the limited sample size, and this approach may not fully account for sex-related differences in body composition and prognostic risk. Although patients with missing key laboratory or imaging data required for CXI assessment were excluded, retrospective data collection may still have introduced unrecognized information bias. The interval between CT acquisition and index hospitalization varied among patients, and some CT examinations may have been performed for unrelated clinical indications, potentially limiting the ability of CXI to fully reflect physiologic status at admission. Finally, although our findings support the potential clinical relevance of CXI in HF, they do not establish causality, and prospective multicenter studies are required before routine clinical implementation can be recommended. The application of strict exclusion criteria was intended to improve cohort homogeneity and minimize potential confounding effects. However, this approach may have reduced the external validity of the study and limited the applicability of the findings to the broader and more heterogeneous HF population. Furthermore, the possibility of selection bias resulting from these exclusions should be acknowledged when interpreting the study outcomes. We acknowledge that our study was retrospective and conducted at a single center, which may limit the generalizability of the findings and introduce potential selection bias. So, these results could not be applicable to all HF patients.

## 5. Conclusions

In conclusion, among patients hospitalized with decompensated heart failure, a low cachexia index was strongly associated with adverse outcomes and showed excellent discriminatory performance for all-cause mortality. The prognostic relevance of CXI likely stems from its ability to integrate three biologically interdependent domains—skeletal muscle depletion, nutritional impairment, and systemic inflammation—into a single clinically interpretable marker. The additional independent association of low hematocrit with mortality further suggests that reduced hematologic reserve accompanies this high-risk phenotype. Taken together, these results support CXI as a promising integrative prognostic marker in hospitalized HF and suggest that it may serve as a potential prognostic marker; however, prospective studies are required before clinical application can be recommended. However, larger prospective and multicenter studies are needed to validate these findings, refine optimal cut-offs, and determine whether CXI-guided risk stratification can improve patient management and outcomes.

## Figures and Tables

**Table 1:** Baseline demographic, clinical parameters of the study population by gender.

Variable	Male (*n* = 65)	Female (*n* = 62)	Total (*n* = 127)	*p* Value
Age (year)	67.43 ± 13.10	73.61 ± 11.60	70.45 ± 12.73	0.006
Atrial fibrillation, *n* (%)	25 (38.5)	32 (51.6)	57 (44.9)	0.136
Pulmonary disease, *n* (%)	19 (29.2)	18 (29.0)	37 (29.1)	0.980
Coronary artery disease, *n* (%)	45 (69.2)	38 (61.3)	83 (65.4)	0.347
PCI, *n* (%)	38 (58.5)	30 (48.4)	68 (53.5)	0.255
CABG *n* (%)	18 (27.7)	5 (8.1)	23 (18.1)	0.004
Pulmonary arterial hypertension *n*, (%)	11 (16.9)	3 (4.8)	14 (11.0)	0.030
Chronic kidney disease, *n* (%)	28 (43.8)	32 (52.5)	60 (48.0)	0.330
Diabetes mellitus, *n* (%)	36 (55.4)	31 (50.0)	67 (52.8)	0.543
Hypertension, *n* (%)	57 (87.7)	59 (95.2)	116 (91.3)	0.135
SMI (cm^2^/m^2^)	48.65 (34.20–54.67)	36.88 (30.20–41.78)	40.76 (31.46–50.87)	˂0.001
Cachexia index	34.62 (19,82–57.91)	32.40 (14.97–48.61)	33.58 (18.13–53.74)	0.300
Low cachexia index	19 (29.2)	22 (35.5)	41 (32.3)	0.451
High cachexia index	46 (70.8)	40 (64.5)	86 (67.7)	
All-cause mortality, *n* (%)	18 (27.7)	19 (30.6)	37 (29.1)	0.714
Number of HF admissions	1 (0–3)	1 (0–4)	1 (0–3)	0.696
Beta-blocker, *n* (%)	54 (83.1)	47 (77.0)	101 (80.2)	0.363
ACEI/ARB, *n* (%)	51 (78.5)	47 (77.0)	98 (77.8)	0.849
MRA, *n* (%)	25 (38.5)	16 (26.2)	41 (32.5)	0.143
SGLT-2 inhibitor, (%)	10 (15.9)	7 (11.5)	17 (13.7)	0.477
Diuretic, *n* (%)	57 (87.7)	44 (72.1)	101 (80.2)	**0.029**
NOAC, *n* (%)	25 (38.5)	28 (45.9)	53 (42.1)	0.398
Ivabradine, *n* (%)	12 (18.5)	4 (6.6)	16 (12.7)	**0.045**

Normally distributed continuous variables are presented as mean ± standard deviation, non-normally distributed variables as median (interquartile range), and categorical variables as frequency (percentage). ACEI: Angiotensin-Converting Enzyme Inhibitor; ARB: Angiotensin Receptor Blocker; CABG: Coronary Artery Bypass Grafting; HF: Heart Failure; NOAC: Non-Vitamin K Antagonist Oral Anticoagulant; MRA: Mineralocorticoid Receptor Antagonist; PCI: Percutaneous Coronary Intervention; SGLT-2: Sodium–Glucose Cotransporter 2; SMI: Skeletal Muscle Index. Values with *p* < 0.05 were considered statistically significant and are indicated in bold.

**Table 2:** Echocardiographic and laboratory parameters of the study population by gender.

Variable	Male (*n* = 65)	Female (*n* = 62)	Total (*n* = 127)	*p* Value
Ejection fraction (%)	30 (25–45)	50 (40–60)	40 (30–50)	**˂** **0.001**
Ejection fraction, *n* (%)				
EF > 50%	12 (18.5)	37 (59.7)	49 (38.6)	**˂** **0.001**
EF 41–49%	9 (13.8)	10 (16.1)	19 (15.0)	
EF < 40%	44 (67.7)	15 (24.2)	59 (46.5)	
LVEDd (mm)	56.27 ± 9.19	49.88 ± 7.88	52.82 ± 9.04	**0.001**
LA (mm)	46.92 ± 6.47	44.21 ± 5.05	45.56 ± 5.93	**0.042**
IVS (mm)	10 (10–12)	11 (10–13)	10 (10–13)	0.151
sPAP (mmHg)	40 (36–50)	42 (30–50)	40 (35–50)	0.343
NT pro-BNP (ng/L)	13,961 ± 10,703	12,923 ± 11,649	13,362 ± 11,162	0.741
BNP (pg/mL)	1517 ± 1130	938 ± 581	1300 ± 988	0.170
WBC (10^9^/L)	7290 (6240–9370)	7905 (5820–9420)	7630 (6180–9420)	0.662
Hemoglobin (g/dL)	11.30 ± 2.00	10.15 ± 1.58	10.74 ± 1.89	**˂** **0.001**
Hematocrit (%)	34.63 ± 5.85	31.10 ± 4.55	32.91 ± 5.53	**˂** **0.001**
Platelet count (10^9^/L)	255,476.92 ± 97,557.21	258,935.48 ± 92,442.43	257,165.35 ± 94,733.36	0.838
Neutrophils (10^9^/L)	5170 (4430–7170)	5835 (4230–7700)	5480 (4370–7770)	0.877
Lymphocytes (10^9^/L)	1240 (1000–1500)	1265 (850–1510)	1240 (900–1510)	0.881
NLR	4.29 (3.25–6.69)	4.32 (3.53–6.32)	4.29 (3.25–6.69)	0.946
Creatinine (mg/dL)	1.10 (10–1.60)	1.40 (10–1.70)	1.20 (10–1.70)	0.254
Urea (mg/dL)	65 (47–79)	65.5 (46–94)	65 (46–88)	0.407
eGFR (mL/dk/1.73 m^2^)	61 (39–78)	38 (29–56)	50 (31–74)	**˂** **0.001**
Sodium (mmol/L)	138 (135–140)	139 (135–140)	138 (135–140)	0.521
Potassium (mmol/L)	4.35 ± 0.68	4.41 ± 0.64	4.38 ± 0.66	0.597
Lactate dehydrogenase (U/L)	268.18 ± 87.48	299.25 ± 130.72	283.85 ± 112.01	0.141
C-reactive protein (mg/L)	11.5 (6–35)	10 (6–44.9)	11 (6–37)	0.927

Normally distributed continuous variables are presented as mean ± standard deviation, non-normally distributed variables as median (interquartile range), and categorical variables as frequency (percentage). BNP: B-type Natriuretic Peptide; EF: Ejection Fraction; eGFR: estimated Glomerular Filtration Rate; IVS: Interventricular; LA: Left Atrium; LVEDd: Left Ventricular End-Diastolic Diameter; NLR: Neutrophil-to-Lymphocyte Ratio; sPAP: Systolic Pulmonary Artery Pressure; WBC: White Blood Cell. Values with *p* < 0.05 were considered statistically significant and are indicated in bold.

**Table 3:** Comparison of clinical characteristics between patients with low and high cachexia index.

Variable	Low CXI Group (*n* = 41)	High CXI Group (*n* = 86)	*p*
Age (years)	72.00 ± 13.54	69.71 ± 12.34	0.345 ^†^
Ejection fraction (EF) *n* (%)			
EF > 50	15 (36.6)	34 (39.5)	0.400 *
EF 41–49	4 (9.8)	15 (17.4)	
EF < 40	22 (53.7)	37 (43.0)	
Ejection fraction	35 (30–55)	45 (30–50)	0.552 ^‡^
Atrial fibrillation	20 (48.8)	37 (43.0)	0.542 *
Pulmonary disease	11 (26.8)	26 (30.2)	0.693 *
Coronary artery disease	28 (68.3)	55 (64.0)	0.631 *
PCI	22 (53.7)	46 (53.5)	0.986 *
CABG	4 (9.8)	19 (22.1)	0.091 *
Pulmonary arterial hypertension	4 (9.8)	10 (11.6)	0.753 *
Chronic kidney disease	21 (52.5)	39 (45.9)	0.490 *
Diabetes mellitus	16 (39.0)	51 (59.3)	**0.032** *
Hypertension	35 (85.4)	81 (94.2)	0.098 *
Beta-blocker	33 (80.5)	68 (80.0)	0.333 *
ACE-I/ARB	30 (73.2)	68 (80.0)	0.388 *
MRA	11 (26.8)	30 (35.3)	0.342 *
SGLT-2 inhibitor	2 (4.9)	15 (18.1)	**0.044** *
Diuretic	32 (78.0)	69 (81.2)	0.680 *
NOAC	17 (41.5)	36 (42.4)	0.925 *
Ivabradine	2 (4.9)	14 (16.5)	0.067 *
LVEDd (mm)	55 (47.5–59)	51 (46–57)	0.148 ^‡^
LA (mm)	45.61 ± 5.14	45.55 ± 6.27	0.966 ^†^
IVS (mm)	11 (10–13)	10 (10–13)	0.708 ^‡^
sPAP (mmHg)	43.5 (32.5–55)	40 (35–45)	0.419 ^‡^
NT pro-BNP (ng/L)	15,825 ± 12,971	12,267 ± 10,268	0.293 ^†^
BNP (pg/mL)	994.40 ± 744.43	1518.07 ± 1104.98	0.207 ^†^
WBC (10^9^/L)	8720 (6730–9420)	6945 (6080–8820)	**0.012** ^‡^
Hemoglobin (g/dL)	10.55 ± 1.97	10.84 ± 1.85	0.421 ^†^
Hematocrit (%)	32.30 ± 5.53	33.19 ± 5.54	0.398 ^†^
Platelet count (10^9^/L)	247,512 ± 117,526	261,767 ± 82,079	0.430 ^†^
Neutrophils (10^9^/L)	6950 (4730–7770)	5235 (4080–7250)	**<0.001** ^‡^
Lymphocytes (10^9^/L)	832 (670–1510)	1190 (1045–1510)	**<0.001** ^‡^
NLR	8.67 (6.14–12.59)	3.69 (2.83–4.61)	**<0.001** ^‡^
Creatinine (mg/dL)	1.26 (0.8–1.8)	1.2 (1.0–1.6)	0.890 ^‡^
Urea (mg/dL)	69 (47–94)	62 (44–81)	0.306 ^‡^
eGFR (mL/dk/1.73 m^2^)	45 (32–74)	52.5 (31–73)	0.826 ^‡^
Sodium (mmol/L)	137 (135–140)	139 (136–140)	0.138 ^‡^
Potassium (mmol/L)	4.39 ± 0.69	4.37 ± 0.65	0.845 ^†^
Lactate dehydrogenase (U/L)	327.69 ± 143.14	263.35 ± 87.85	**0.004** ^†^
C-reactive protein (mg/L)	33 (7–68)	8 (6–24)	**<0.001** ^‡^
SMI (cm^2^/m^2^)	30 (26.7–34.8)	45.0 (38.2–54.4)	**<0.001** ^‡^
Number of HF admissions	4 (3–5)	0.5 (0–1)	**<0.001** ^‡^
All-cause mortality	33 (80.5)	4 (4.7)	**<0.001** *

Normally distributed continuous variables are presented as mean ± standard deviation, non-normally distributed variables as median (interquartile range), and categorical variables as frequency (percentage). * *p*-value from Fisher’s exact test or chi-square test, ^†^ *p*-value from Student’s *t*-test, ^‡^ *p*-value from Mann–Whitney U test. ACEI: Angiotensin-Converting Enzyme Inhibitor; ARB: Angiotensin Receptor Blocker; BNP: B-type Natriuretic Peptide; CABG: Coronary Artery Bypass Grafting; EF: Ejection Fraction; eGFR: estimated Glomerular Filtration Rate; HF: Heart Failure; IVS: Interventricular; LA: Left Atrium; LVEDd: Left Ventricular End-Diastolic Diameter; NLR: Neutrophil-to-Lymphocyte Ratio; Non-Vitamin K Antagonist Oral Anticoagulant; MRA: Mineralocorticoid Receptor Antagonist; PCI: Percutaneous Coronary Intervention; SGLT-2: Sodium–Glucose Cotransporter 2; SMI: Skeletal Muscle Index; sPAP: Systolic Pulmonary Artery Pressure; WBC: White Blood Cell. Values with *p* < 0.05 were considered statistically significant and are indicated in bold.

**Table 4:** Association of demographic, clinical, and laboratory characteristics with mortality.

Variable	Survivor *n* (%)	Mortality *n* (%)	*p*
Female	47 (52.2)	18 (48.6)	0.714
Male	43 (47.8)	19 (51.4)	
Age < 70	44 (48.9)	17 (45.9)	0.763
Age > 70	46 (51.1)	20 (54.1)	
Ejection fraction			
EF > 50	34 (37.8)	15 (40.5)	0.938
EF 41–49	14 (15.6)	5 (13.5)	
EF < 40	42 (46.7)	17 (45.9)	
Atrial fibrillation	39 (43.3)	18 (48.6)	0.584
Pulmonary disease	28 (31.1)	9 (24.3)	0.444
Coronary artery disease	58 (64.4)	25 (67.6)	0.737
PCI	47 (52.2)	21 (56.8)	0.642
CABG	17 (18.9)	6 (16.2)	0.722
Pulmonary arterial hypertension	10 (11.1)	4 (10.8)	0.961
Chronic kidney disease	40 (44.4)	20 (57.1)	0.202
Diabetes mellitus	51 (56.7)	16 (43.2)	0.169
Hypertension	83 (92.2)	33 (89.2)	0.581
Beta-blocker	71 (78.9)	30 (83.3)	0.736
ACE-I/ARB	72 (80.0)	26 (72.2)	0.343
MRA	30 (33.3)	11 (30.6)	0.764
SGLT-2 inhibitor	13 (14.8)	4 (11.1)	0.591
Diuretic	73 (81.1)	28 (77.8)	0.672
NOAC	36 (40.0)	17 (47.2)	0.458
Ivabradine	13 (14.4)	3 (8.3)	0.352
LVEDd < 53	35 (55.6)	11 (42.3)	0.255
LVEDd > 53	28 (44.4)	15 (57.7)	
LA < 45	34 (60.7)	10 (45.5)	0.221
LA > 45	22 (39.3)	12 (54.5)	
IVS < 10	3 (5.2)	3 (13.6)	0.199
IVS > 10	55 (94.8)	19 (86.4)	
sPAP < 40	17 (32.7)	8 (38.1)	0.660
sPAP > 40	35 (67.3)	13 (61.9)	
Pro-BNP < 10,509	20 (54.1)	6 (40.0)	0.358
Pro-BNP > 10,509	17 (45.9)	9 (60.0)	
WBC < 7630	51 (56.7)	12 (32.4)	0.013
WBC > 7630	39 (43.3)	25 (67.6)	
BNP < 1038	9 (52.9)	3 (42.9)	0.653
BNP > 1038	8 (47.1)	4 (57.1)	
Hemoglobin < 10.65	40 (44.9)	23 (62.2)	0.078
Hemoglobin > 10.65	49 (55.1)	14 (37.8)	
Hematocrit (%) < 33	37 (41.1)	23 (62.2)	**0.031**
Hematocrit (%) > 33	53 (58.9)	14 (37.8)	
Platelet count < 238,000	43 (47.8)	19 (51.4)	0.714
Platelet count > 238,000	47 (52.2)	18 (48.6)	
Neutrophils < 5480	54 (60.0)	9 (24.3)	**<0.001**
Neutrophils > 5480	36 (40.0)	28 (75.7)	
Lymphocytes < 1240	33 (36.7)	28 (75.7)	**<0.001**
Lymphocytes > 1240	57 (63.3)	9 (24.3)	
NLR < 4.29	59 (65.6)	4 (10.8)	**<0.001**
NLR > 4.29	31 (34.4)	33 (89.2)	
Creatinine < 1.2	41 (45.6)	16 (43.2)	0.812
Creatinine > 1.2	49 (54.4)	21 (56.8)	
Urea < 65	47 (52.2)	16 (43.2)	0.358
Urea > 65	43 (47.8)	21 (56.8)	
eGFR < 50	42 (46.7)	20 (54.1)	0.449
eGFR > 50	48 (53.3)	17 (45.9)	
Sodium < 138	37 (41.1)	19 (51.4)	0.291
Sodium > 138	53 (58.9)	18 (48.6)	
Potassium < 4.4	44 (48.9)	19 (51.4)	0.801
Potassium > 4.4	46 (51.1)	18 (48.6)	
Lactate dehydrogenase < 260	41 (51.9)	14 (41.2)	0.296
Lactate dehydrogenase > 260	38 (48.1)	20 (58.8)	
C-reactive protein < 11	51 (58.0)	11 (29.7)	**0.004**
C-reactive protein > 11	37 (42.0)	26 (70.3)	
SMI < 40	29 (32.2)	33 (89.2)	**<0.001**
SMI > 40	61 (67.8)	4 (10.8)	
Number of HF admissions < 1	43 (47.8)	0 (0.0)	**<0.001**
Number of HF admissions > 1	47 (52.2)	37 (99.7)	

*p*-values were calculated using Fisher’s exact test or Pearson’s chi-square test. ACEI: Angiotensin-Converting Enzyme Inhibitor; ARB: Angiotensin Receptor Blocker; BNP: B-type Natriuretic Peptide; CABG: Coronary Artery Bypass Grafting; EF: Ejection Fraction; eGFR: estimated Glomerular Filtration Rate; HF: Heart Failure; IVS: Interventricular; LA: Left Atrium; LVEDd: Left Ventricular End-Diastolic Diameter; NLR: Neutrophil-to-Lymphocyte Ratio; Non-Vitamin K Antagonist Oral Anticoagulant; MRA: Mineralocorticoid Receptor Antagonist; PCI: Percutaneous Coronary Intervention; SGLT-2: Sodium–Glucose Cotransporter 2; SMI: Skeletal Muscle Index; sPAP: Systolic Pulmonary Artery Pressure; WBC: White Blood Cell. Values with *p* < 0.05 were considered statistically significant and are indicated in bold.

**Table 5:** Univariate and multivariate logistic regression analysis for the prediction of all-cause mortality.

	Univariate LR		Stepwise Multivariate LR	
	OR (95 CI)	*p* Value	OR (95 CI)	*p* Value
Gender (Male)	0.867 (0.403–1.864)	0.714		
Age > 70	1.125 (0.522–2.424)	0.763		
Low CXI	84.562 (23.833–300.036)	**<0.001**	98.968 (23.227–421.691)	**<0.001**
Ejection fraction < 40	1	0.938		
41–49	0.810 (0.247–2.656)	0.727		
>50	0.917 (0.401–2.101)	0.839		
Atrial fibrillation (1)	1.239 (0.575–2.670)	0.584		
Pulmonary disease (1)	0.712 (0.297–1.705)	0.582		
Coronary artery disease (1)	1.149 (0.510–2.590)	0.737		
PCI (1)	1.201 (0.556–2.595)	0.642		
CABG (1)	0.831 (0.299–2.308)	0.723		
Pulmonary arterial hypertension (1)	0.970 (0.284–3.312)	0.961		
Chronic kidney disease (1)	1.667 (0.758–3.665)	0.204		
Diabetes mellitus (1)	0.583 (0.269–1.262)	0.171		
Hypertension (1)	0.696 (0.191–2.535)	0.582		
Beta-blocker (1)	1.147 (0.436–3.016)	0.782		
ACE-I/ARB (1)	0.650 (0.266–1.589)	0.345		
MRA (1)	0.880 (0.382–2.025)	0.764		
SGLT-2 inhibitor (1)	0.721 (0.218–2.381)	0.592		
Diuretic (1)	0.815 (0.316–2.100)	0.672		
NOAC (1)	1.342 (0.616–2.923)	0.459		
Ivabradine (1)	0.538 (0.144–2.016)	0.358		
LVED > 53	1.705 (0.677–4.291)	0.258		
LA > 45	1.855 (0.685–5.021)	0.224		
IVS > 10	0.345 (0.064–1.859)	0.216		
sPAP > 40	0.789 (0.275–2.265)	0.660		
ProBNP > 10,509	1.765 (0.522–5.969)	0.361		
WBC > 7630	2.724 (1.218–6.092)	**0.015**	1.763 (0.445–6.986)	0.420
BNP > 1038	1.500 (0.254–8.844)	0.654		
Hemoglobin < 10.65	0.497 (0.227–1.089)	0.081		
Hematocrit (%) < 33	2.353 (1.072–5.164)	**0.033**	3.979 (1.001–16.341)	**0.048**
Platelet count > 238,000	0.867 (0.403–1.864)	0.714		
Neutrophils > 5480	4.67 (1.72–11.043)	**<0.001**		
Lymphocytes < 1240	5.374 (2.263–12.760)	**<0.001**		
NLR > 4.29	15.702 (5.097–48.368)	**<0.001**		
Creatinine > 1.2	1.098 (0.508–2.375)	0.812		
Urea > 65	1.435 (0.664–3.101)	0.359		
eGFR < 50	1.345 (0.624–2.898)	0.450		
Sodium > 138	0.661 (0.306–1.427)	0.292		
Potassium > 4.4	0.906 (0.421–1.949)	0.801		
Lactate dehydrogenase > 260	1.541 (0.684–3.476)	0.297		
C-reactive protein > 11	3.258 (1.432–7.414)	**0.005**	2.208 (0.555–8.785)	0.261
SMI < 40	17.353 (5.617–53.609)	**<0.001**		
Number of HF admissions > 1	12.717 (0.001–20.964)	0.997		

(1) Indicates presence. Bold values are statistically significant (*p* < 0.05). CI: confidence interval; OR: odds ratio; LR: logistic regression. ACEI: Angiotensin-Converting Enzyme Inhibitor; ARB: Angiotensin Receptor Blocker; BNP: B-type Natriuretic Peptide; CABG: Coronary Artery Bypass Grafting; EF: Ejection Fraction; eGFR: estimated Glomerular Filtration Rate; HF: Heart Failure; IVS: Interventricular; LA: Left Atrium; LVEDd: Left Ventricular End-Diastolic Diameter; NLR: Neutrophil-to-Lymphocyte Ratio; Non-Vitamin K Antagonist Oral Anticoagulant; MRA: Mineralocorticoid Receptor Antagonist; PCI: Percutaneous Coronary Intervention; SGLT-2: Sodium–Glucose Cotransporter 2; SMI: Skeletal Muscle Index; sPAP: Systolic Pulmonary Artery Pressure; WBC: White Blood Cell. Values with *p* < 0.05 were considered statistically significant and are indicated in bold.

## Data Availability

The data presented in this study are available from the corresponding author upon reasonable request.

## Abbreviations

HF	Heart failure
CXI	Cachexia index
SMI	Skeletal muscle index
NLR	Neutrophil-to-lymphocyte ratio
CT	Computed tomography
CCU	Coronary care unit
LVEF	Left ventricular ejection fraction
ROC	Receiver operating characteristic
AUC	Area under the curve
CI	Confidence interval
